# Anticonvulsant Profiles of Three Hemorphin-4 Analogs with Rhodamine B in Mice

**DOI:** 10.3390/ph18050673

**Published:** 2025-05-01

**Authors:** Jana Tchekalarova, Miroslav Rangelov, Ivan Iliev, Nadezhda Todorova, Tsveta Stoyanova, Lian Nedelchev, Petar Todorov

**Affiliations:** 1 Institute of Neurobiology, Bulgarian Academy of Sciences, 1113 Sofia, Bulgaria; tzafti@abv.bg; 2 Department of Organic Chemistry, University of Chemical Technology and Metallurgy, 1756 Sofia, Bulgaria; lian_n@yahoo.com (L.N.); pepi_37@abv.bg (P.T.); 3 Institute of Organic Chemistry with Centre of Phytochemistry, Bulgarian Academy of Sciences, 1113 Sofia, Bulgaria; marangelov@gmail.com; 4 Institute of Experimental Morphology, Pathology and Anthropology with Museum, Bulgarian Academy of Sciences, Acad. G. Bonchev Str., bl. 25, 1113 Sofia, Bulgaria; taparsky@abv.bg; 5 Institute of Biodiversity and Ecosystem Research, Bulgarian Academy of Sciences, 1113 Sofia, Bulgaria; nadeshdahr@gmail.com; 6 Institute of Optical Materials and Technology, Bulgarian Academy of Sciences, Acad. G. Bonchev Str. bl. 109, P.O. Box 95, 1113 Sofia, Bulgaria

**Keywords:** hemorphin, seizure test, PTZ kindling, cytotoxicity and phototoxicity, docking analysis, mice

## Abstract

**Background/Objectives**: Hemorphins, considered to be bioactive atypical oligopeptides, are products of hemoglobin metabolism. Recently, our team reported the synthesis and characterization of three N-modified analogs of hemorphin-4 (H4) with rhodamine B (Rh). In the present study, the Rh-1, Rh-2, and Rh-3 compounds were intracerebroventricularly infused at doses of 1, 2.5, 5, and 10 µg/5 µL, respectively, and evaluated for their antiseizure activity in 6-Hz and maximal electroshock (MES) tests and in a pentylenetetrazol (PTZ)-induced kindling model in mice. Phenytoin and diazepam were used as the reference drugs. The role of opioid receptors (ORs) underlying their mechanism of action was also evaluated in silico and pharmacologically. **Results**: The three Rh-H4 compounds showed a good safety profile at a concentration of 100 µg/mL in the mouse embryonic fibroblasts. They suppressed psychomotor seizures and seizure spreading as follows: Rh-1 at doses of 5 and 10 µg/5 µL, Rh-2 at the highest dose, and Rh-3 at doses of 1–10 µg/5 µL, respectively. Administered at doses of 5 µg/5 µL (Rh-1 and Rh-3) and 10 µg/5 µL (Rh-2), the compounds suppressed clonic seizures in the kindled mice comparable to the reference drug diazepam. A combination of selective delta (DOR), kappa (KOR), and mu (MOR) OR antagonists with the highest doses of the Rh-1, Rh-2, and Rh-3 compounds was used to elucidate the possible role of ORs in the underlying mechanism related to their protective activity against seizure spread. Only the selective DOR antagonist, natrindole, suppressed the effect of the Rh-1 peptide analog on seizures. The OR antagonist naloxone prevented the antiseizure activity of Rh-1 in the kindled mice. The results of docking analysis also showed the model-specific interaction of the three Rh-H4 compounds with the OR. **Conclusions**: Our results suggest that the antiseizure activity of Rh-1 is mediated by the OR, and in particular by the DOR, while the mechanism underlying the antiseizure effect of Rh-3 is more complex and may involve other receptors.

## 1. Introduction

Epilepsy is a neurological disorder with symptoms closely related to convulsive or non-convulsive seizures due to an imbalance between inhibition and excitation in the brain structures, characterized by a low seizure threshold. Approximately 50 million people worldwide live with epilepsy, with a slightly higher prevalence in men than in women [1]. The drugs available on the market are effective in suppressing the main symptoms of epilepsy, but most of them can cause side effects, including headaches, nausea, fatigue, memory problems, and mood changes. In addition, about 30 percent of patients are resistant to anticonvulsant treatment. Research into the discovery of novel molecules to create drugs with potential anticonvulsant activity is ongoing and crucial to improving the treatment options for people with this condition. Scientists’ efforts to develop drugs for the treatment of epilepsy involve various approaches, including the design and synthesis of compounds targeting specific seizure suppression mechanisms, such as the neurotransmitter or neuromodulatory systems involved in neuronal excitation and inhibition, e.g., fast Na^+^ and Ca^2+^ channels. In preclinical studies, novel compounds are screened for potential anticonvulsant activity using animal models of epilepsy, and these may be modifications of existing drugs or entirely new chemical entities. These studies investigate their efficacy, safety, and plausible side effects.

Hemorphins, the products of hemoglobin metabolism, are considered to be bioactive atypical oligopeptides of from four to ten amino acids. Their mechanism of action, related to the activation of opioid receptor (OR) subtypes, predisposes them to be considered as atypical endogenous peptides. There is a group of endogenous N-terminal opioid tetrapeptides with similar amino acid sequences [2,3]. The tetrapeptide hemorphin-4 (H4) has been isolated from a variety of sources, including the enzymatic digestion of casein and hemoglobin. The Tyr-Pro dipeptide sequence found at the N-terminus of these tetrapeptides has been identified as a specific agonist of mu OR (MOR). It is fascinating that even small changes in the structure of hemorphins, such as positional changes or alterations in the incorporated groups, can lead to significant shifts in their activity and affinity [3]. These findings suggest that the exploration of different modifications could lead to the development of novel hemorphin analogs with potentially enhanced therapeutic properties or improved pharmacokinetic profiles.

The literature data, including ours, indicate that hemorphin-like tetrapeptides possess a wide range of properties, such as analgesic, anti-inflammatory, antiseizure, and antihypertensive [4,5,6,7,8]. These findings highlight the diverse physiological effects of hemorphin-like tetrapeptides and indicate their potential importance in modulating various physiological processes. Our team has extensive experience in the design, synthesis, and characterization of hemorphin analogs [4,5]. The solid-phase peptide synthesis (SPPS) technique is effective for the production of N-terminal-modified molecules. This chemistry allows for the design and targeted introduction of different amino acid residues and the incorporation of many pharmacophores into a single molecule, often with a broader and synergistic mechanism of action.

In addition, our team has recently reported the synthesis and characterization of three N-modified rhodamine (Rh) B analogs of H4 [9]. They were analyzed by UV-Vis and fluorometric methods. The compound consisting of a hydrophobic Abu-Tyr-Pro-Trp-Thr-CONH2 amino acid sequence with Rh B attached at the N-terminus (Rh-3) was found to be the most active H-4 analog with potent antiviral activity. The aim of this work was to gather evidence on the importance of different aminoalkyl residues in newly synthesized hybrid compounds to understand their biological and pharmacological properties in potential drug applications. In the present study, the aforementioned novel rhodamine-linked H4s were evaluated in vivo for their antiseizure activity in these well-known acute seizure tests used as a first screen to select the most effective dose: the maximal electroshock (MES) test and the 6-Hz psychomotor seizure test. In addition, the most effective dose of the three compounds, Rh-1, Rh-2, and Rh-3, was characterized in a pentylenetetrazole (PTZ)-induced model of epilepsy in mice. Phenytoin and diazepam were used as the reference drugs for comparative analysis. The role of the ORs underlying their mechanism of action was also investigated by docking analysis and a pharmacological approach. Three selective antagonists of the delta OR (DOR), the kappa OR (KOR), and the MOR were used.

## 2. Results

### 2.1. N-Modified Analogs of Hemorphin-4 with Rhodamine B

**Figure 1:**
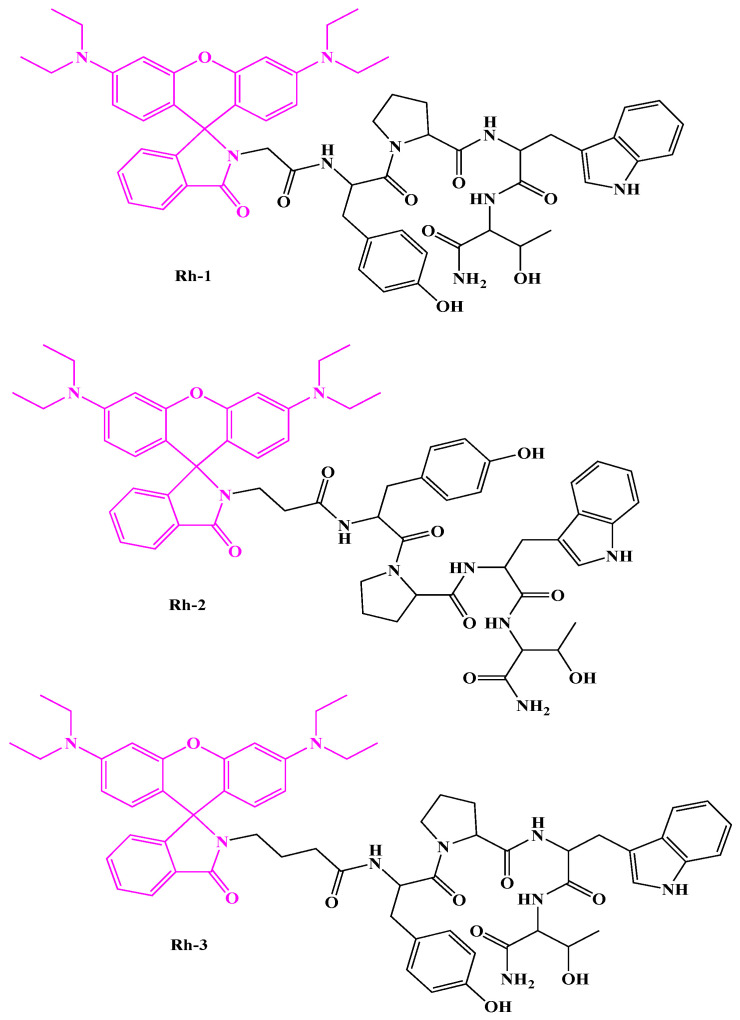
Chemical structures of investigated new rhodamine (Rh) B-conjugated hemorphin-4 (H4) analogs.

Rhodamine B was attached directly to peptidyl resin, yielding the desired peptide compounds. The handling of the amino acid scaffold can be considered a potentially important process in both bio-organic and medicinal chemistry investigations, as well as in the production of new drugs and materials. The synthesized biopeptide molecules Rh B-Gly-Tyr-Pro-Trp-Thr-NH2 (Rh-1), Rh B—Ala-Tyr-Pro-Trp-Thr-NH2 (Rh-2), and Rh B-γ-Abu-Tyr-Pro-Trp-Thr-NH2 (Rh-3) show potential biological activity and differ from natural H-4 by having more methylene groups (from one to three) between the Rh B moiety and the N-side and the amino acid backbone. Shown in Figure 1.

### 2.2. Cytotoxicity/Phototoxicity in BALB 3T3 Cells

**Figure 2:**
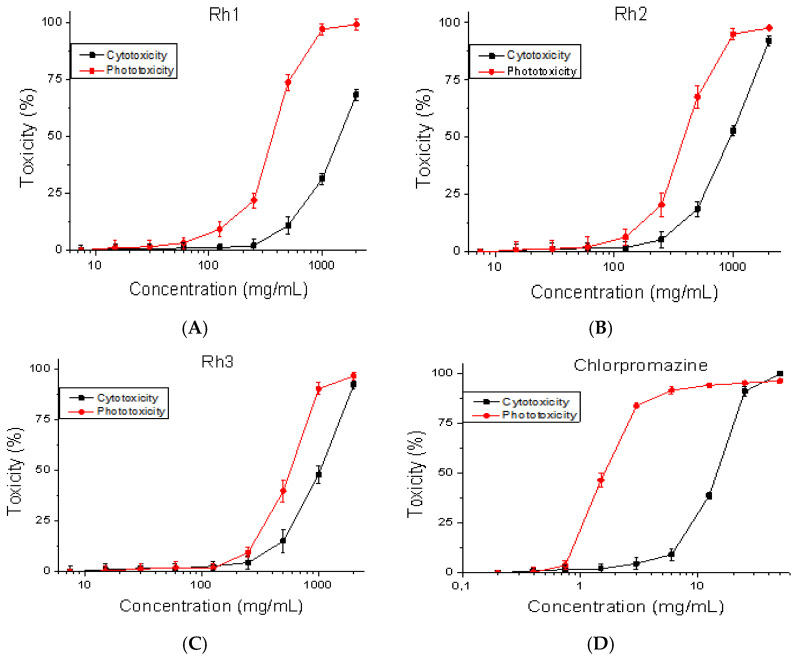
Dose–response curves for cytotoxicity/phototoxicity of compounds in BALB/3T3 cells. (**A**) Rh-1, (**B**) Rh-2, (**C**) Rh-3, and (**D**) Chlorpromazine (positive control). Values are means ± SD from three independent experiments, n = 6.

The compounds were studied for cytotoxicity/phototoxicity by a 3T3 NRU-test. The cells were incubated with the test substances for 24 h at 37 °C, 5% CO_2_, and 95% humidity. Cytotoxicity expressed in % relative to the negative control was determined. Dose–response dependence was observed for all the compounds. The results are shown in Figure 2. At a concentration of 100 µg/mL, no cytotoxic effect was observed on the test substances.

The CC_50_ values (50% cytotoxic concentration) were calculated by nonlinear regression analysis (Table 1). The compound Rh-1 has the lowest toxicity (CC_50_ = 1422.21 ± 44.87 μg/mL). The CC_50_ values can be used to calculate the PIF (photo-irritation factor) for each test compound according to this formula: PIF = CC_50_ (−Irr)/CC_50_ (+Irr). The PIF shows us the probability that the test substance may cause a phototoxic effect (PIF < 2 not phototoxic, 2 ≤ PIF < 5 probable phototoxicity, and PIF ≥ 5 phototoxic) (Table 2). The calculated PIF factor for the substances Rh-1 and Rh-2 is higher than two. This indicates that these substances are probably weakly phototoxic.

### 2.3. Muscle Strength and Motor Coordination

Muscle strength, as assessed using grip strength apparatus and motor coordination measured in the rotarod test, were not affected by the three Rh-conjugated compounds administered at the highest dose (10 µg/5 µL) (Table 3).

The data are presented as mean muscle strength (in Newtons ± S.D.) in the mice subjected to the grip strength test. The compounds were infused intracerebroventrivularly (icv) 15 min prior to the tests at a dose of 10 µg/5 µL (n = 6–8).

### 2.4. Seizure Tests

**Figure 3:**
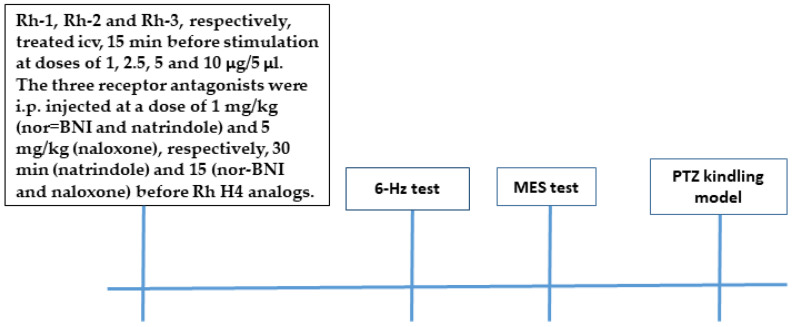
Experimental steps timeline.

A timeline of the three experimental tests used to investigate the anticonvulsant effects of the Rh H4 analogs is shown in Figure 3.

#### 2.4.1. The 6-Hz Test

**Figure 4:**
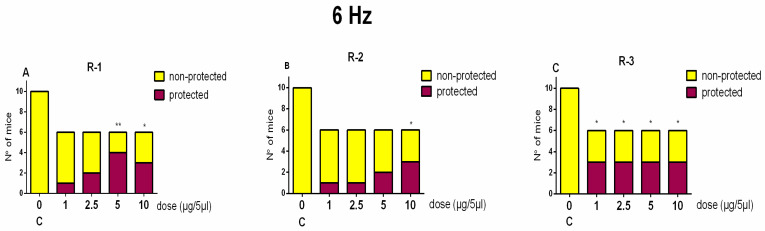
Ratio of protected vs. non-protected mice against psychomotor seizures induced by 6-Hz corneal stimulation in control mice for (C), Rh-4 (**A**), Rh-2 (**B**), and Rh-3 (**C**), respectively, treated icv 15 min before stimulation at doses of 1, 2.5, 5, and 10 µg/5 µL (n = 6–8). Fisher’s exact test: * *p* < 0.05 and ** *p* < 0.01 vs. C, respectively.

The Rh1 compound showed activity against psychomotor seizures induced by 6-Hz corneal stimulation at the two highest doses of 5 and 10 µg/mouse (Fisher exact test: *p* < 0.01 and *p* < 0.05 vs. control, respectively) (Figure 4). The Rh-2 compound showed efficacy against psychomotor seizures only at the highest dose of 10 µg/mouse (*p* < 0.05 vs. control), while the third Rh-3 compound showed potent antiseizure efficacy at the four doses tested (*p* < 0.05 vs. control).

#### 2.4.2. Maximal Electroshock Seizure (MES) Test

**Figure 5:**
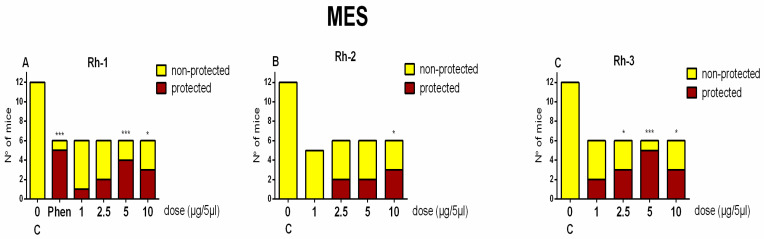
Ratio of protected vs. non-protected mice against the MES-induced tonic–clonic seizures in control mice for (C) Rh-4 (**A**), Rh-2 (**B**), and Rh-3 (**C**), respectively, treated icv 15 min before stimulation at doses of 1, 2.5, 5, and 10 µg/mouse (n = 6–8). Fisher’s exact test: * *p* < 0.05, and *** *p* < 0.001 vs. C, respectively.

Like in the 6-Hz test, the Rh-1 compound exhibited antiseizure activity in the MES test at the two highest doses of 5 and 10 µg/mouse (*p* < 0.01 and *p* < 0.001 vs. C, respectively) (Figure 5A). The Rh-2 compound showed activity against tonic–clonic seizures at the highest dose of 10 µg/mouse (*p* < 0.001 vs. C) (Figure 5B). The Rh-3 compound had the highest potency to suppress seizure spread in the MES test at the three doses of 2.5, 5, and 10 µg/mouse (*p* < 0.05 and *p* < 0.001 vs. C, respectively) (Figure 5C). Moreover, the antiseizure activities of the Rh-1 and Rh-3 compounds were comparable to that of the reference drug phenytoin (Phen) (Figure 5A,C).

**Figure 6:**
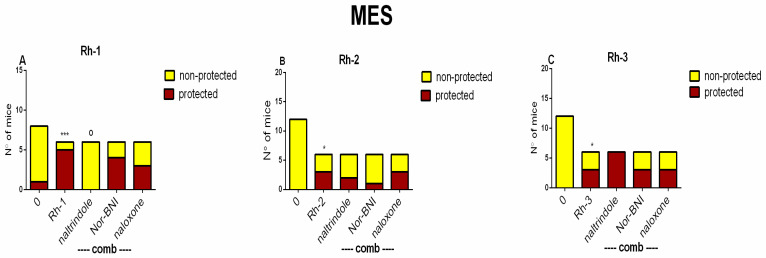
Ratio of protected vs. non-protected mice against MES-induced tonic–clonic seizures in controls (0), Rh-1, combination of natrindole + Rh-1, nor-BNI, and naloxone, respectively (**A**); 0, Rh-2, combination of natrindole + Rh-2, nor-BNI, and naloxone, respectively (**B**); and 0, Rh-3, combination of natrindole + Rh-3, nor-BNI, and naloxone, respectively (**C**). The three receptor antagonists were i.p. injected at doses of 1 mg/kg (nor-BNI and natrindole) and 5 mg/kg (naloxone), respectively, 30 min (natrindole) and 15 min (nor-BNI and naloxone) before icv infusion of Rh-1, Rh-2, and Rh-3, given at doses of 5 µg/5, 10 µg/5 µL, and 5 µg/5, respectively (n = 6–8). Fisher’s exact test: ^o^ *p* < 0.015, natrindole + Rh-1 vs. Rh-1. * *p* < 0.05 and *** *p* < 0.001 vs. C, respectively, ^o^ *p* = 0.05 vs. Rh-1.

A combination of the selective OR antagonists of the DOR, the KOR, and the MOR with the highest doses of the Rh-1, Rh-2, and Rh-3 compounds, respectively, was used to elucidate the possible role of ORs in the underlying mechanism related to the protective activity of H-4 analogs against MES-induced seizure-spreading. The selective antagonist of the DOR, natrindole, suppressed the antiseizure effect of the Rh-1 peptide analog (*p* < 0.015 vs. Rh-1) (Figure 6A). The selective antagonists of the KOR and the MOR, respectively, were unable to reverse the effect of the Rh-1 compound (*p* > 0.05 vs. Rh-1). The selective antagonists of the DOR, the KOR, and the MOR did not affect the antiseizure efficacy of the Rh-2 compound though the KOR antagonist. Nor-BNI, demonstrated the tendency to reverse the protective effect of Rh-1 at a dose of 5 µg/5 µL (Figure 6B). Also, the three OR antagonists did not block the antiseizure effect of the Rh-3 compound (Figure 6C).

#### 2.4.3. Pentylenetetrazol (PTZ) Kindling Model of Epilepsy

**Figure 7:**
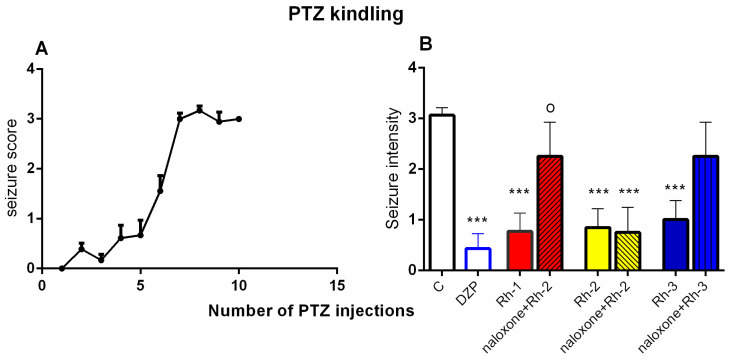
Seizure intensity measured from 1st to 10th injection of subconvulsive dose of PTZ (35 mg/kg, i.p.) (**A**). Seizure intensity in fully kindled mice (after 10th PTZ injection) in controls (**B**), diazepam-treated group (DZP) (5 mg/kg, i.p.), and Rh-1 (5 µg/mouse, icv), naloxone (1 mg/kg, i.p.) + Rh-1, Rh-2 (10 µg/mouse, icv), naloxone (1 mg/kg, i.p.) + Rh-2, Rh-3 (5 µg/mouse, icv), and naloxone (5 mg/kg, i.p.) + Rh-3 (n = 8–13) groups. One-way ANOVA [F8,79 = 4.029, *p* = 0.0009]; *** *p* < 0.001; DZP, Rh-1, Rh-2, and Rh-3 vs C; ^o^ *p* < 0.05, naloxone + Rh-1 vs. Rh-1.

The effect of the three Rh B-conjugated H-4 analogs were tested on the fully kindled mice by repetitive PTZ injection. The Rh-1 and Rh-3 compounds were applied at a dose of 5 µg/mouse, while the Rh-2 compound was infused at the highest dose of 10 µg/mouse based on its efficacy against 6-Hz and MES-induced seizures, respectively. To assess the plausible opioid-related mechanism underlying the Rh B-conjugated analogs’ activity, they were also studied in a combination with the non-selective OR antagonist naloxone. Seizure susceptibility increased after the seventh PTZ injection at a subconvulsive dose of 35 mg/kg and reached a plateau after the ninth injection (stages 3–4) (Figure 7A). The three Rh analogs suppressed the clonic seizures induced by the subconvulsive dose of PTZ on the fully kindled mice (*p* < 0.001; Rh-1, Rh-2, and Rh-3 vs. C), the effect of which was comparable to that of the reference drug diazepam (DZP) (*p* < 0.001, DZP vs. C) (Figure 7B). While naloxone inhibited the antiseizure effect of Rh-1 (*p* < 0.05, naloxone + Rh-1 vs. Rh-1), it showed a tendency to reverse the antiseizure effect of Rh-3 and ineffectively reversed this effect in combination with Rh-2.

### 2.5. Molecular Docking Analysis

**Figure 8:**
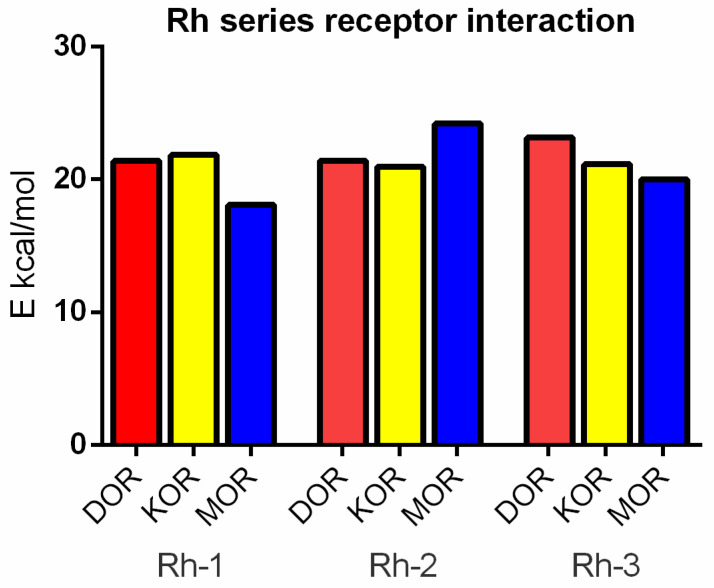
Interaction energies between DOR (in red), KOR (in yellow), and MOR (in blue) with rhodamine B-conjugated hemorphin-4 analogs. More positive value of interaction energies means better binding between ligand and receptor.

The interaction energy of Rh-1 showed similar binding capacities for the DOR and the KOR, but a minimal capacity for the MOR (Figure 8). The opposite result is shown for the interaction of Rh-2, where the best binding was shown with the MOR, whereas Rh-3 binding for the KOR was slightly diminished than that with the DOR.

**Figure 9:**
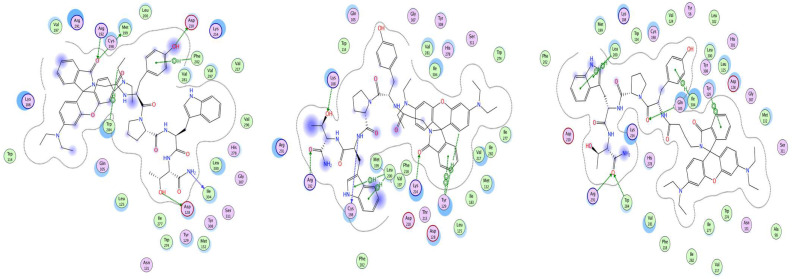
Interaction map of best poses of Rh-1 (**left**), Rh-2 (**center**), and Rh-3 (**right**) ligands with DOR.

**Figure 10:**
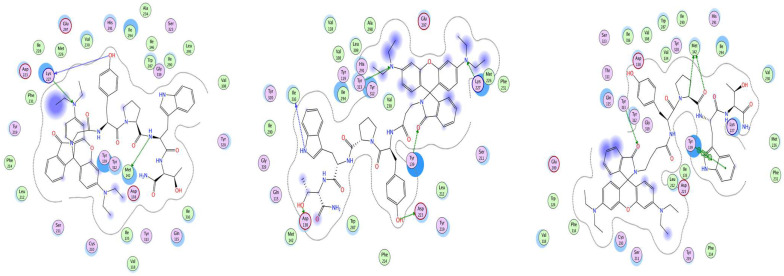
Interaction map of best poses of Rh-1 (**left**), Rh-2 (**center**), and Rh-3 (**right**) ligands with KOR.

**Figure 11:**
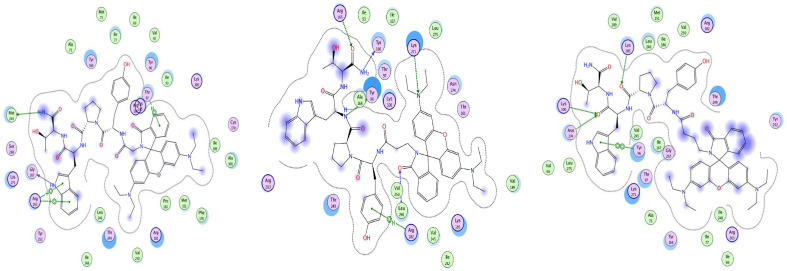
Interaction map of best poses of Rh-1 (**left**), Rh-2 (**center**), and Rh-3 (**right**) ligands with MOR.

Figure 9, Figure 10 and Figure 11 show the best poses of interaction between the newly synthesized ligands and the KOR, the DOR, and the MOR. We have used color coding, where green represents the lipophilic amino acids; pink represents the polar amino acids; the red, circled amino acids are acidic; and the blue, circled amino acids are basic. The green arrows illustrate the side-chain interactions of the amino acids. The involvement of the backbone in the interaction is indicated by a blue arrow, starting from the donor of the H-bond, with the arrowhead pointing towards the acceptor of the H-bond.

The blue halo around the ligand atoms outlines their exposure to the solvent, with the same feature, but for the amino acids of the receptor visible as blue circles. The grey dotted line represents the proximity contour of the pocket.

**Figure 12:**
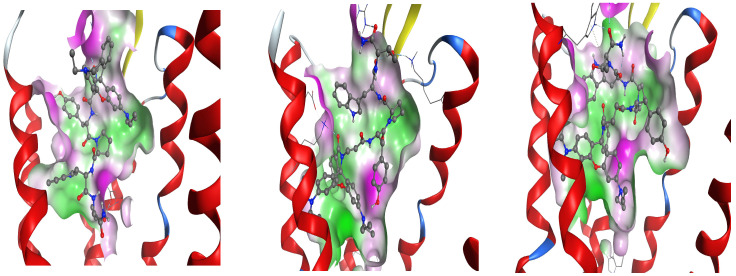
Surface of receptor active site pockets in case of DOR for Rh-1 (**left**), Rh-2 (**center**), and Rh-3 (**right**), colored according to site lipophilicity (green) and hydrophilicity (magenta).

**Figure 13:**
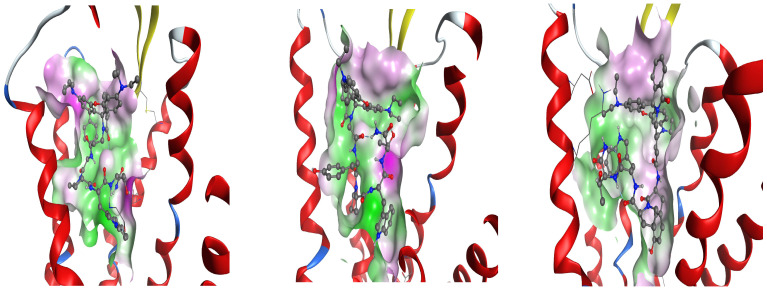
Surface of receptor active site pockets in case of KOR for Rh-1 (**left**), Rh-2 (**center**), and Rh-3 (**right**), colored according to site lipophilicity (green) and hydrophilicity (magenta).

The difference between the volumes of active site regions of the DOR, the KOR, and the MOR, respectively, can be seen in Figure 12 and Figure 13.

## 3. Discussion

In this study, the antiseizure properties of three novel H4 analogs conjugated to Rh B (Rh-1, Rh-2, and Rh-3) were evaluated in two acute screening tests routinely used in experimental practice, with different mechanisms of action, the 6-Hz psychomotor seizure test and the MES seizure spreading test. In addition, the three Rh H4 compounds were evaluated at their most potent doses in a chronic kindling model induced by repeated subconvulsive doses of PTZ in mice. While Rh-1 and Rh-3 showed almost similar efficacies against the psychomotor seizures and tonic–clonic seizures induced by MES, the Rh-2 compound had an antiseizure effect only at the highest dose of 10 µg/mouse. The selective antagonists of the DOR, the KOR, and the MOR were pretreated with the three Rh H4 compounds administered at the highest dose of 10 µg/mouse in the MES test and the PTZ kindling model, respectively. Our results suggest that Rh-1 is the most potent antiseizure compound, whose activity is mediated by the ORs, and in particular by the DOR.

The PTZ kindling model is widely used in the study of partial complex epilepsy [10]. In fully kindled mice, a subconvulsive dose of PTZ induces a clonic seizure due to increased brain susceptibility that mirrors the process of epileptogenesis, a central component of epilepsy development. According to Kupferberg [11], this model has proved valuable for studying the mechanisms underlying epileptic activity and for testing potential antiepileptic drugs. It is particularly useful for studying the mechanisms underlying the antiseizure activity of novel compounds.

Endogenous opioid peptides are involved in the regulation of hippocampal excitability via direct actions on the principal neurons, where the ORs are expressed on divergent cells with inhibitory function, such as GABAergic interneurons, parvalbumin positive basket cells, and somatostatin-expressing interneurons [12]. Opioid peptides have ambiguous effect on seizure susceptibility, depending on the affinity of different ORs [13]. G-protein-coupled ORs are found in the brain and throughout the body. They play a key role in pain modulation, reward, and addiction mechanisms. Ongoing research suggest that DORs may play a modulatory role in seizure susceptibility, either as proconvulsants or anticonvulsants depending on the context and the neurotransmitter systems involved [13]. The latter may be due to the influence of DORs on GABAergic (inhibitory) and glutamatergic (excitatory) neurotransmission, where increased GABAergic activity may reduce seizure susceptibility. In addition, DORs may interact with several other neurotransmitter systems, including the endocannabinoid and neuropeptide systems, which may also influence seizure activity. The KOR has attracted increasing interest due to its predominance in pain-related neurons, making it a promising candidate for drug therapies targeting psychiatric disorders [14]. As can be seen from the results of this experiment, all the ligands studied have a different amino acid pattern to the KOR, whose interaction is the object of this study. The docking shows the involvement of receptor sites, which are known to be functional in other natural and man-made ligands. The anchoring residue Asp138, which is conserved in all opioid receptors and plays a critical role in KOR activation [15,16], is one of the residues involved in interaction with Rh ligands. It has been noted that Asp138 and Tyr 139 are both classified as conserved residues that have been shown to be involved in interaction with Rh ligands [17]. Met142 has been shown to resides in specific residues in the orthosteric pockets that interact with Rh-1 and Rh-3. Furthermore, the mutational analysis of Met142 has been shown to alter receptor activation [18]. It is evident that a number of other factors are involved in the interaction of the optimal positions of our ligands with the receptor, including Tyr139, Tyr313, Ile316, and Lys227. They have been reported to influence signal transduction, the type of G-protein coupling, and the future fate of a signal by affecting distance between the transmembrane parts of the receptor [18,19]. The interaction of the KOR parts with ligands occurs primarily through surface contact. However, Tyr139, Tyr313, and Lys227 also play an important role as proton donors, while Asp138, Met142, Asp223, Lys227, and Ile316 act as proton acceptors. It has been shown that certain residues are involved, some of which have been suggested to be important for the specificity of a receptor. For example, Trp284 has been identified as a key player in this process [20]. Another residue from the extracellular loop (ECL), where the major determinant of selectivity is located, is Arg291. The interaction of our ligands with the regions of the DOR that have been identified as critical for ligand specificity [17,20] is a key finding of this study. Rh-1 was found to interact with Asp128, which is located at the base of the DOR pocket. In contrast, Rh-2 and Rh-3 were observed to form H-bonds with the ECL2 region. Arg291 primarily engages with the ligand through surface contact, acting as a hydrogen bond donor with its peptide bond. In contrast, Lys108 and Leu200 predominantly interact as hydrogen bond donors with their amide bond. Asp128, Trp284, and Met199 have been shown to interact with the DOR as hydrogen bond acceptors. In the context of amino acids, it has been shown that Tyr96 and Lys100, which have been identified as the key elements in MOR interactions with our ligands, play a significant role in the specific methadone-induced activation of the receptor [20]. This specific active conformation of the MOR differs from those described for classical opioid drugs, and thus provides probable hypotheses for the nature of the subsequent fate of the signal from the MOR. MOR activation has been shown to induce the translocation of TM6 to TM3, accompanied by the disruption of the H-bond between Arg165 and Thr279. Arg165 is an important conserved part of the TM3 helix, together with Tyr166 [17]. This pair of residues is known to be involved in the active state of the MOR. Therefore, it is not unexpected that there is ligand interaction between these two residues [21].

The results of this study showed that the three H4 compounds conjugated to Rh were able to protect the fully kindled mice against the clonic seizures induced by the subconvulsive dose of PTZ. These three novel H4 analogs conjugated to Rh B showed comparable antiseizure activities to those of the reference drugs phenytoin and DZP, respectively, which act by different mechanisms and have distinct pharmacological profiles. Thus, while phenytoin is able to limit seizure spread in the MES test by stabilizing the inactive state of voltage-gated Na^+^ channels on neurons, DZP can moderate overexcited neurons by increasing the affinity of the GABA_A_ receptor for GABA [22,23].

Docking analysis suggests that the mechanism of action of Rh-1 against seizure onset is mediated by the DOR and the KOR, with the minor involvement of the MOR. In addition, other pharmacological studies showed that the antiseizure activity of Rh-1 was suppressed by the competitive DOR antagonist, natrindole, in an MES test and by naloxone in fully kindled mice. The non-selective and competitive antagonist of ORs, naloxone, has the highest affinity for the MOR, followed by a lower affinity for the DOR, and then for the KOR. This finding suggests that DOR activation plays a dominant role in the antiseizure effect of this compound. Conversely, interaction with the MOR, as determined by the docking results, was only significant for Rh-2. It is hypothesized that the effects of the second compound, Rh-2, are mediated by the MOR based on the pharmacological data showing that this compound has antiseizure activity at the highest dose used in tests with a different mechanism of action. For the third Rh conjugated H4 compound, Rh-3, the pharmacological and docking data suggest that its antiseizure activity is predominantly mediated by the DOR.

The intersection of opioid agonists (particularly for KORs and DORs) and antiseizure activity is a fascinating and evolving area of research. Opioids are best known for their analgesic properties. However, there is increasing interest in their potential effects on seizure activity. Nevertheless, opioid tolerance remains a major challenge in the field of opioid pharmacology. The activation of the MOR is the primary driver of tolerance via receptor internalization, anenylyl cyclase upregulation, and other such mechanisms. The strategies to overcome tolerance and other side effects have included the development of selective DOR agonists that avoid rapid internalization and a lack of tolerance, the targeting of DOR-MOR heteromers that could improve opioid efficacy without increasing tolerance, and so on. DOR agonists have been reported to possess antidepressant and anxiolytic effects in rodent models [24]. When used in a controlled manner, opioids have the ability to disrupt inflammatory cycles in epilepsy, thereby preserving neuronal function and reducing seizures [25]. The ability of endogenous peptides, enkephalin, and enkephalin agonists to suppress seizure propagation in a rat MES test has been demonstrated. This suppression is mediated by the activation of MORs and DORs, resulting in the hyperpolarization of neurons in the central nervous system [26]. The interaction of our ligands with all the ORs is characterized by high diversity in the pattern of the amino acids involved. The involvement of all the three ORs is not unexpected, as they are approximately 70% similar in their transmembrane regions, with the differences concentrated mainly in the extracellular and intracellular loops.

## 4. Materials and Methods

### 4.1. Safety Testing

Mouse embryonic fibroblasts (BALB/3T3 clone A31, ATCC: CCL-163™) were used to perform the cytotoxicity and phototoxicity tests. The cell line was obtained from the American Type Cultures Collection (ATCC, Manassas, VA, USA). A safety test was performed using OECD Guidelines for the Testing of Chemicals, Section 4, Test No. 432. Cell viability was measured using the BALB 3T3 Neutral Red Uptake assay [27]. The cells were plated at 1 × 10^4^ cells/well in 96-well microplates. After 24 h of incubation, the test substances were added at various concentrations (7.5–2000 μg/mL). In the phototoxicity test, 96-well plates were irradiated (+Irr) with a dose of 2.4 J/cm^2^ using a solar light simulator Helios-iO (SERIC Ltd., Tokyo, Japan). Cytotoxicity is presented as % relative to the negative control.

### 4.2. Animals

Two-month-old male ICR mice (20–30 g), purchased by the vivarium of the Institute of Neurobiology, BAS, were left without treatment for a week after arrival. They were accommodated in standard conditions before the experiments as follows: transparent cages (8–10 mice per cage), a strict diurnal regime of 12:12 light/dark exposure, temperature (22 ± 1 °C), and access to animal pellets and tap water ad libitum. The procedures were conducted in agreement with the Declaration of Helsinki on the care and use of animals (DHEW publication, NHI 80-23) and the EC Directive 2010/63/EU on animal experimentation. The experimental design was approved by the Bulgarian Food Safety Authority (license number: 354/2023).

### 4.3. Drugs and Treatment

The Rh B-conjugated H-4 analogs (Rh-1, Rh-2, and Rh-3) were prepared using SPPS by Fmoc (9-fluorenylmethoxy-carbonyl) chemistry as reported in our recent study [4]. They were freshly dissolved in 1% DMSO before each test and were infused icv (5 µL/ventricle) at a rate of 1 l/30 s and at doses of 1, 2.5, 5, and 10 µg/mouse depending of the test as described previously [4]. Briefly, a midline scalp incision was made in ether-anaesthetized mice. A small hole was drilled in the scalp to implant a cannula over the right lateral ventricle (AP: −0.3 mm; L: 1 mm). Each drug was infused 24 h after surgery using a 10-µL Hamilton^®^ 28-gauge syringe (the tip of the syringe’s blunt needle was terminated in the ventricle 2.75 mm below the dura mater). The controls were similar, except for the administration of a vehicle instead of a drug. In the MES test, selective antagonists of the KOR (nor-binaltorphimine, (nor-BNI), 1 mg/kg), the DOR (naltrindole, 1 mg/kg), and naloxone (5 mg/kg) were administered intraperitoneally (i.p.) at a volume of 10 mL/kg body weight 15 min (naltrindole) or 30 min (nor-BNI) before Rh B-conjugated H4 analogs were administered (10 µg). In the fully kindled mice, competitive antagonists of the MOR, the KOR, and the DOR (naloxone, 5 mg/kg) (FOT, Sofia, Bulgaria) dissolved in saline were pretreated i.p. at a volume of 10 mL/kg body weight 15 min before Rh B-conjugated H-4 analog administration (10 µg). Seizure intensity was assessed 15 min after icv infusion.

### 4.4. Grip Strength and Rotarod Test

The effects of the three Rh-conjugated H4 analogs on neuromuscular tone and muscle strength were evaluated using grip strength apparatus (BIOSEB, Cedex, Vitrolles, France) by pulling the animals backwards at the tail. Strength was calculated as the average of at least three measurements in Newtons (N).

Possible motor relaxation was assessed using the rotarod test. The mice were placed on the rotating rod (3.2 cm in diameter at a speed 10 rpm), and the criterion for the absence of motor deficit was the ability to remain stable on the moving rod for at least 1 min out of three trials. From six to eight mice were used in each group.

### 4.5. Tests for AntiSeizure Activity

#### 4.5.1. The 6-Hz Test

Before each stimulation, a drop of anesthesia solution was applied to the eyes of each mouse. Activity against psychomotor seizures was assessed by using a stimulus (6 Hz, 32 mA, 3 s, 0.2 ms duration monopolar rectangular pulses) via corneal electrodes as described in our previous study [4]. Immediately after stimulation, the treated mice were placed in a cage for assessment of their behavior. The criterion for antiseizure activity was accepted if stereotypic responses, such as eye blinking, hyperlocomotion, Straub’s tail, and clonic seizures, did not disappear after 10 s of observation. From six to eight mice per each group were used.

#### 4.5.2. Maximal Electroshock Seizure Test

Generalized tonic–clonic seizures were provoked by a corneal electrical stimulus (60 Hz, 50 mA, 0.2 s) in the mice. The replacement of the tonic extension of hind limbs by forelimb tonic extension or clonic seizures was accepted as antiseizure activity. From six to eight mice per each group were used.

#### 4.5.3. Pentylenetetrazol Kindling Model

The procedure to develop a kindling state in the mice was performed by the intraperitoneal (i.p.) injection of PTZ (Sigma-Aldrich, St. Louis, MO, USA, 35 mg/kg, dissolved in saline) on Monday, Wednesday, and Friday for up to 10–12 injections, with the criterion of at least three consecutive stage 3–5 seizures (forelimb clonus progressing to clonic convulsion with the loss of righting reflex). With each PTZ injection, the seizure phases occurred gradually. After each PTZ injection, the mice were observed for 30 min for the onset, duration, and latency of clonic convulsions. Seizure intensity was scored using a modified Racine scale: 0 = normal response; 1 = single myoclonic jerks and/or facial clonus; 2 = more than 3 myoclonic jerks; 3 = clonic forelimb convulsion without postural loss; 4 = clonic convulsion with rearing; 5 = clonic convulsion with the loss of righting reflex; and 6 = tonic–clonic convulsion. Following the PTZ treatment protocol, 90% of the mice were fully anesthetized, and after 48 h the animals were divided into eight groups (n = 8–13), a control group; a DZP-treated group (reference group); three groups treated with Rh-1, Rh-2, and Rh-3 analogs at an effective dose in the MES test; and the three Rh-B-conjugated H-4 analogs combined with the OR antagonist naloxone to evaluate their potential mechanism of action. The mice that failed to burn were not used to test drug effects. The animals were observed and scored for seizure onset for 0.5 h after being placed in Plexiglas cages.

### 4.6. Docking Analysis

As a way to suggest a possible molecular mechanism of action and interaction for our modified rhodamine ligands and receptors from the opioid group—delta, kappa, and mu, respectively—we have selected to work with proteins with a known crystallized 3D structure with good resolution. Based on this criterion, further molecular modeling was conducted with the following protein structures obtained from the RCSB Protein Data Bank (RCSB PDB): 8F7S for the DOR, 8F7W for the KOR, and 5C1M for the MOR. For consistency, we have decided to use the PDB entries of human opioid receptors from a single study published by Wang et al. [28]. The initial selected receptors were in a complex with ligands.

The existing slight structural properties in selected PDB were amended, with non-protein species being eliminated. The MOE software suite was employed for achieving the correct protonation state at 7.0 pH, 300 K, and salt concentration 0.1 M/L, using physiological entries by applying Labute algorithm [29].

The modeled receptors have to be specific for mice, with consideration of the test animals; therefore, modification for in silico mutation was performed on the crystallized human structures. The UniProt database entries for Mus musculus were utilized as follows: DOR-P32300, KOR-P33534, and MU-P42866.

This docking study was accomplished using the protonated state of our ligands at 7 pH, where the AMBER12EHT force field with the application of the LowModeMD methodology was chosen for the generation of a conformational library of the receptor residues, where only those of them that meet the criterion conformational energy value of up to 5 kcal/mol higher than the lowest one, were further selected for this work.

The Edelsbrunner site finder algorithm [30] was used as it is implemented in the MOE2020 software suite for the adequate description of the active site cavity, which is necessary for the fast and adequate placement of ligands in the docking stage

The specificity of the OR active state, namely being pretty tight, necessitated the application of the Alpha PMI docking method (MOE2020) in all of the selected pockets.

The scoring function of the resultant poses was applied using the London dG (MOE2020) function, which evaluates the free energy of the binding of the ligand in a given pose by accounting for the geometric imperfections of hydrogen bonds and the desolvation energy of atoms. London dG approximates the average gain/loss of rotational and translational entropy and the loss of flexibility of the ligand.

From the pool of the 100 poses with the best London dG for every ligand for every pocket, further refinement was conducted with the Induced Fit methodology, with a cutoff of 6A for the ligand using the AMBER12EHT force field and the Generalized Born solvation model. The obtained results were rescored with the GBVI/WSA dG (MOE2020) scoring function. Further analysis was performed based on best 30 poses using the above-described procedure.

Where a stable complex between a ligand and a receptor is achievable, the calculated binding energy is negative, which is the case for interaction between all our ligands and receptors. Therefore, for the convenience of result interpretation, we define the negative value of the binding energies as “interaction energies” between them.

### 4.7. Statistical Analysis

The CC50 values were calculated using nonlinear regression analysis (GraphPad Prism 8 Software). The data from the safety test are averaged from three independent experiments ± SD. The data from the animal tests are expressed as mean ± SEM. Fisher’s exact test (two-tailed) was used to assess protection in the 6-Hz test and the MES test. The data from the safety test and the PTZ kindling model were estimated by one-way ANOVA of variance. Post hoc Bonferroni’s or the Dunn test was applied in case of factor significance. Statistical significance was accepted at *p* < 0.05.

## 5. Conclusions

The pharmacological data and docking analysis suggest that the two Rh-conjugated H4 analogs, Rh-1 and Rh-3, are potent antiseizure compounds. The activity of Rh-1 is mediated by the OR, and in particular by the DOR, while the mechanism underlying the antiseizure effect of Rh-3 is more complex and may involve other receptors. The results obtained are consistent with known models of GPCR (part of which are ORs), which state that the different chemotypes of ligand exhibit different and unique types of binding and manifested outcomes. We report an individual pattern of receptor activation of our ligands, with both shared and unique interacting residues, supporting the idea of a non-singular mode of interaction and activation of ORs.

## Figures and Tables

**Table 1:** Some chemical parameters of the N-modified analogs of H4 with Rh B.

Code	Peptides	Molecular Formula	Molecular Weight
Rh-1	Rh B-Gly-Tyr-Pro-Trp-Thr-NH_2_	C_59_H_67_N_9_O_9_	1046.23
Rh-2	Rh B-β-Ala-Tyr-Pro-Trp-Thr-NH_2_	C_60_H_69_N_9_O_9_	1060.26
Rh-3	Rh B-γ-Abu-Tyr-Pro-Trp-Thr-NH_2_	C_61_H_71_N_9_O_9_	1074.29

**Table 2:** Cytotoxicity/phototoxicity in BALB 3T3 cells. Mean CC_50_ and photo-irritation factor.

Compounds	Mean CC_50_ ± SD (µg/mL)		PIF *
	−Irr	+Irr **	
Rh-1	1422.21 ± 44.87	364.62 ± 15.90	3.9
Rh-2	944.81 ± 37.50	386.16 ± 12.10	2.4
Rh-3	1027.75 ± 70.85	573.92 ± 32.76	1.8
Chlorpromazine ***	14.53 ± 0.325	1.621 ± 0.205	8.9

* PIF (photo-irritation factor), PIF < 2 not phototoxic, 2 ≤ PIF < 5 probable phototoxicity, and PIF ≥ 5 phototoxic. ** Ir—irradiation; *** Chlorpromazine (positive control).

**Table 3:** The effects of the N-modified analogs of H4 with Rh B on neuromuscular tone in the grip strength test and motor coordination in the rotarod test in the mice.

Group/Treatment	Dose (mg/kg), i.p.	Grip Strength (N)	Rotarod Test N/F
Control (saline)	0	2.08 ± 0.48	0/8
Rh-1	10	1.23 ± 0.28	1/7
Rh-2	10	2.34 ± 0.36	1/7
Rh-3	10	1.046 ± 0.41	2/6

## Data Availability

All data generated or analyzed during this study are included in this article. Further inquiries can be directed to the corresponding authors.

## Abbreviations

The following abbreviations are used in this manuscript:

CC_50_	values 50% cytotoxic concentration
DZP	diazepam
DOR	delta
ECL	extracellular loop
Phen	Phenytoin
H4	hemorphin-4
icv	intracerebroventrivularly
i.p.	intraperitoneally
KOR	kappa
MES	maximal electroshock
MOR	mu
OR	opioid receptors
PIF	photo-irritation factor
PTZ	pentylenetetrazol
Rh	rhodamine
SPPS	solid-phase peptide synthesis
